# Instituting Change

**DOI:** 10.1371/journal.pbio.0040224

**Published:** 2006-06-13

**Authors:** Hemai Parthasarathy

## Abstract

MIT helps its researchers comply with the NIH policy on submitting published research to PubMed Central.

Academic success depends so heavily on publication records because funding agencies recognize that the research they sponsor is only as valuable as its impact on science and society. As an increasing number of funding agencies realize the need to enhance impact by reducing restrictions on access to research articles, academic and research institutions have an opportunity to play a key role in securing change.

The United States' National Institutes of Health (NIH) was among the first funding agencies to explicitly recommend the public archiving of the research it supports. As of May 2, 2005, NIH asked their grantees to deposit research articles in the National Library of Medicine's PubMed Central (PMC) archive within 12 months of acceptance by a journal [ 1]. A year later, just 4% of its grantees are complying.

A study by the Publishing Research Consortium designed to “assess understanding of, and compliance with, NIH Public Access Policy” [ 2] found that although 85% of NIH funded authors had heard of this policy, just 18% of surveyed investigators said that they knew a lot about it. Most authors were confused about which version of their paper they were allowed to upload given the copyright agreements they had signed with publishers.

But funding agencies, individual investigators, and publishers are not the only parties who have a stake in the scientific literature. Ann Wolpert, Director of Libraries at the Massachusetts Institute of Technology (MIT), understands that academic and research institutions also have a vested interest in preserving and promoting the research literature that their faculty creates. That is why she and the Vice President for Research and Associate Provost, Alice Gast, are asking publishers to allow MIT researchers to publish their work using a copyright amendment of MIT's design. The amendment allows the author and institution non-exclusive rights to reuse, reproduce, and archive their published research articles in digital repositories (see Text S1). Although this amendment is now available on the MIT Web site and has been distributed to the MIT faculty, Wolpert is taking the extra step of directly contacting the 30 major publishers with whom MIT faculty publish to finalize appropriate language that will best “accommodate the interests of the academy and those of the publishers,” while supporting the implementation of the NIH policy.

According to Wolpert, the MIT copyright amendment grew out of the faculty's double-edged response to the NIH policy recommendation: despite “a philosophical groundswell of agreement,” researchers found the demands of work too overwhelming to overcome the logistical obstacles of acting on their beliefs. Focus groups with faculty and researchers generated a series of recommendations to make compliance easier. One important recommendation was to resolve any conflict between copyright agreements and archiving.

At the stage when a manuscript is (finally!) accepted for publication, the last thing a researcher wants to do is to fight over a copyright agreement, especially when this might cause a publication delay. The purpose of creating this amendment and the subsequent direct discussions with publishers is to bring the institution into the negotiations on the side of the author. Faculty, of course, are pleased to have this potential burden lifted. Although its use is not required, the recommended copyright amendment has the endorsement of several academic groups at MIT, including the Faculty Policy Committee, department heads, and Academic Council.

Institutional interest in reasonable copyright transfer is not unprecedented; indeed as part of the US government, NIH employees do not have copyright over the materials they produce and thus cannot sign publishers' licensing agreements (but curiously, this does not apply to government supported extramural grantees). Institutions may have more power than they realize, in this regard, and many are starting to take notice. Indeed, several equally high-profile research institutions have contacted MIT for further information about the actions they have taken to promote the NIH policy among their faculty. While a clear copyright policy will not solve the other problems associated with the NIH policy, such as who will deposit the research articles in PMC, it is clearly an important component.

PLoS believes in immediate and full access to the fruits of research funded by the public or in the name of public good. However, we also recognize the value of careful peer review, a diversity of journals, and the need to preserve the positive aspects of the current publishing infrastructure. We are one part of a large set of interested parties that shape the scientific enterprise: funding agencies, institutions, investigators, and publishers all have a part to play in the evolution of science and scientific communication. Institutions like MIT that look to this future in the spirit of advancing scientific knowledge and discovery by taking on new, active, and positive roles will be integral to its success.

## Supporting Information

Text S1 MIT Copyright Amendment (15 KB PDF).
