# Supplementary material for: Instituting Change
Source: PLoS Biol. 2006 Jun 13;4(6):e224. doi: 10.1371/journal.pbio.0040224 (PMC1475700; doi:10.1371/journal.pbio.0040224)
Supplement: Text S1 — (15 KB PDF). [file pbio.0040224.sd001.pdf]

AMENDMENT TO PUBLICATION AGREEMENT

1. THIS Amendment hereby modifies the attached Publication Agreement concerning the following Article:

   ____
   (manuscript title)

   ____
   (journal name)

2. The parties to the Publication Agreement and to this Amendment are:

   ____ (corresponding author),

   and

   ____, (the Publisher).

3. The parties agree that wherever there is any conflict between this Amendment and the Publication Agreement, the provisions of this Amendment are paramount and the Publication Agreement shall be construed accordingly.

4. Notwithstanding any terms in the Publication Agreement to the contrary and in addition to the rights retained by Author or licensed by Publisher to Author in the Publication Agreement and any fair use rights of Author, Author and Publisher agree that the Author shall also retain the following rights:

   a. The Author shall, without limitation, have the non-exclusive right to use, reproduce, distribute, create derivative works including update, perform, and display publicly, the Article in electronic, digital or print form in connection with the Author's teaching, conference presentations, lectures, other scholarly works, and for all of Author's academic and professional activities.

   b. Once the Article has been published by Publisher, the Author shall also have all the non-exclusive rights necessary to make, or to authorize others to make, the final published version of the Article available in digital form over the Internet, including but not limited to a website under the control of the Author or the Author's employer or through any digital repository, such as MIT's DSpace or the National Library of Medicine's PubMed Central database.

   c. The Author further retains all non-exclusive rights necessary to grant to the Author's employing institution the non-exclusive right to use, reproduce, distribute, display, publicly perform, and make copies of the work in electronic, digital or in print form in connection with teaching, digital repositories, conference presentations, lectures, other scholarly works, and all academic and professional activities conducted at the Author's employing institution.

5. **Final Agreement.** This Amendment and the Publication Agreement, taken together, constitute the final agreement between the Author and the Publisher with respect to the publication of the Article and allocation of rights under copyright in the Article. Any modification of or additions to the terms of this Amendment or to the Publication Agreement must be in writing and executed by both Publisher and Author in order to be effective.

AUTHOR	PUBLISHER
______________________________	______________________________
(corresponding author on behalf of all authors)	
______________________________	______________________________
Date	Date

MIT Authors:

Please fax a copy of the agreement to 617-253-8894.
Direct any questions to *amend-cip@mit.edu*
